# Magnesium as an emerging bioactive material for orthopedic applications: bedside needs lead the way from innovation to clinical translation

**DOI:** 10.1093/rb/rbaf032

**Published:** 2025-04-26

**Authors:** Ningze Zhang, Qida Zhang, Hongwei Shao, Zhengming Shan, Jiankun Xu, Wenxue Tong, Ronald Man Yeung Wong, Ling Qin

**Affiliations:** Musculoskeletal Research Laboratory, Department of Orthopaedics & Traumatology, The Chinese University of Hong Kong, Hong Kong SAR 999077, China; Innovative Orthopaedic Biomaterial and Drug Translational Research Laboratory, Li Ka Shing Institute of Health Sciences, The Chinese University of Hong Kong, Hong Kong SAR 999077, China; Musculoskeletal Research Laboratory, Department of Orthopaedics & Traumatology, The Chinese University of Hong Kong, Hong Kong SAR 999077, China; Innovative Orthopaedic Biomaterial and Drug Translational Research Laboratory, Li Ka Shing Institute of Health Sciences, The Chinese University of Hong Kong, Hong Kong SAR 999077, China; Musculoskeletal Research Laboratory, Department of Orthopaedics & Traumatology, The Chinese University of Hong Kong, Hong Kong SAR 999077, China; Innovative Orthopaedic Biomaterial and Drug Translational Research Laboratory, Li Ka Shing Institute of Health Sciences, The Chinese University of Hong Kong, Hong Kong SAR 999077, China; Musculoskeletal Research Laboratory, Department of Orthopaedics & Traumatology, The Chinese University of Hong Kong, Hong Kong SAR 999077, China; Innovative Orthopaedic Biomaterial and Drug Translational Research Laboratory, Li Ka Shing Institute of Health Sciences, The Chinese University of Hong Kong, Hong Kong SAR 999077, China; Musculoskeletal Research Laboratory, Department of Orthopaedics & Traumatology, The Chinese University of Hong Kong, Hong Kong SAR 999077, China; Innovative Orthopaedic Biomaterial and Drug Translational Research Laboratory, Li Ka Shing Institute of Health Sciences, The Chinese University of Hong Kong, Hong Kong SAR 999077, China; Joint Laboratory of Chinese Academic of Science and Hong Kong for Biomaterials, The Chinese University of Hong Kong, Hong Kong SAR 999077, China; Musculoskeletal Research Laboratory, Department of Orthopaedics & Traumatology, The Chinese University of Hong Kong, Hong Kong SAR 999077, China; Innovative Orthopaedic Biomaterial and Drug Translational Research Laboratory, Li Ka Shing Institute of Health Sciences, The Chinese University of Hong Kong, Hong Kong SAR 999077, China; Joint Laboratory of Chinese Academic of Science and Hong Kong for Biomaterials, The Chinese University of Hong Kong, Hong Kong SAR 999077, China; Department of Orthopaedics & Traumatology, The Chinese University of Hong Kong, Hong Kong SAR 999077, China; Musculoskeletal Research Laboratory, Department of Orthopaedics & Traumatology, The Chinese University of Hong Kong, Hong Kong SAR 999077, China; Innovative Orthopaedic Biomaterial and Drug Translational Research Laboratory, Li Ka Shing Institute of Health Sciences, The Chinese University of Hong Kong, Hong Kong SAR 999077, China; Joint Laboratory of Chinese Academic of Science and Hong Kong for Biomaterials, The Chinese University of Hong Kong, Hong Kong SAR 999077, China

**Keywords:** magnesium, degradable and non-degradable orthopedic biomaterials and implants, innovation and clinical translation

## Abstract

With the rapid increase in population aging, the number of surgical operations in orthopedics is expected to increase. The gap between the materials applied in clinical orthopedics and materials in discovery and research is obvious due to regulatory requirements for biosafety and treatment efficacy. For the bedside needs, it is important to overcome hurdles by achieving impactful innovation and clinical translation of orthopedic materials. Magnesium (Mg), as an emerging bioactive material, is one of the vital components of the human body and mainly stored in the musculoskeletal system as either a matrix component or an intracellular element for the homeostasis of various physiological functions. However, the degradation and biomechanical performance limit the applications of Mg. This review aims to explore the current challenges and future directions of Mg for clinical translation and provide an update on biomaterials used in orthopedics, factors driving orthopedic innovation, physiology of magnesium ions (Mg^2+^) and its potential clinical applications. To achieve orthopedic application, modification of the performance of Mg as implantable metals and function of the degradation products of Mg *in vivo* are described. For the clinical needs of treating the steroid-associated osteonecrosis (SAON), Mg screws and Mg-based composite porous scaffolds (Mg/PLGA/TCP: magnesium/poly(lactic-co-glycolic acid) (PLGA)/tricalcium phosphate (TCP)) have been developed, but the challenges of Mg-based implants in load-bearing skeletal sites still exist. To utilize the beneficial biological effects of Mg degradation and overcome the weakness in mechanical stability for fracture fixation, the concept of developing Mg/titanium (Ti) hybrid orthopedic implants is reported, where the Ti component provides effective mechanical support while the inclusion of Mg component potentially optimizes the biomechanical properties of Ti component and facilitate bone healing. This review provides a reference frame for the translation of novel materials and promotes the development of innovative orthopedic biomaterials for clinical applications.

## Graphical abstract

**Figure:**
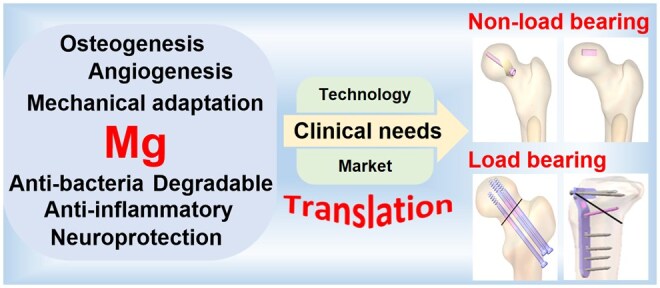

## Introduction

**Figure 1.**
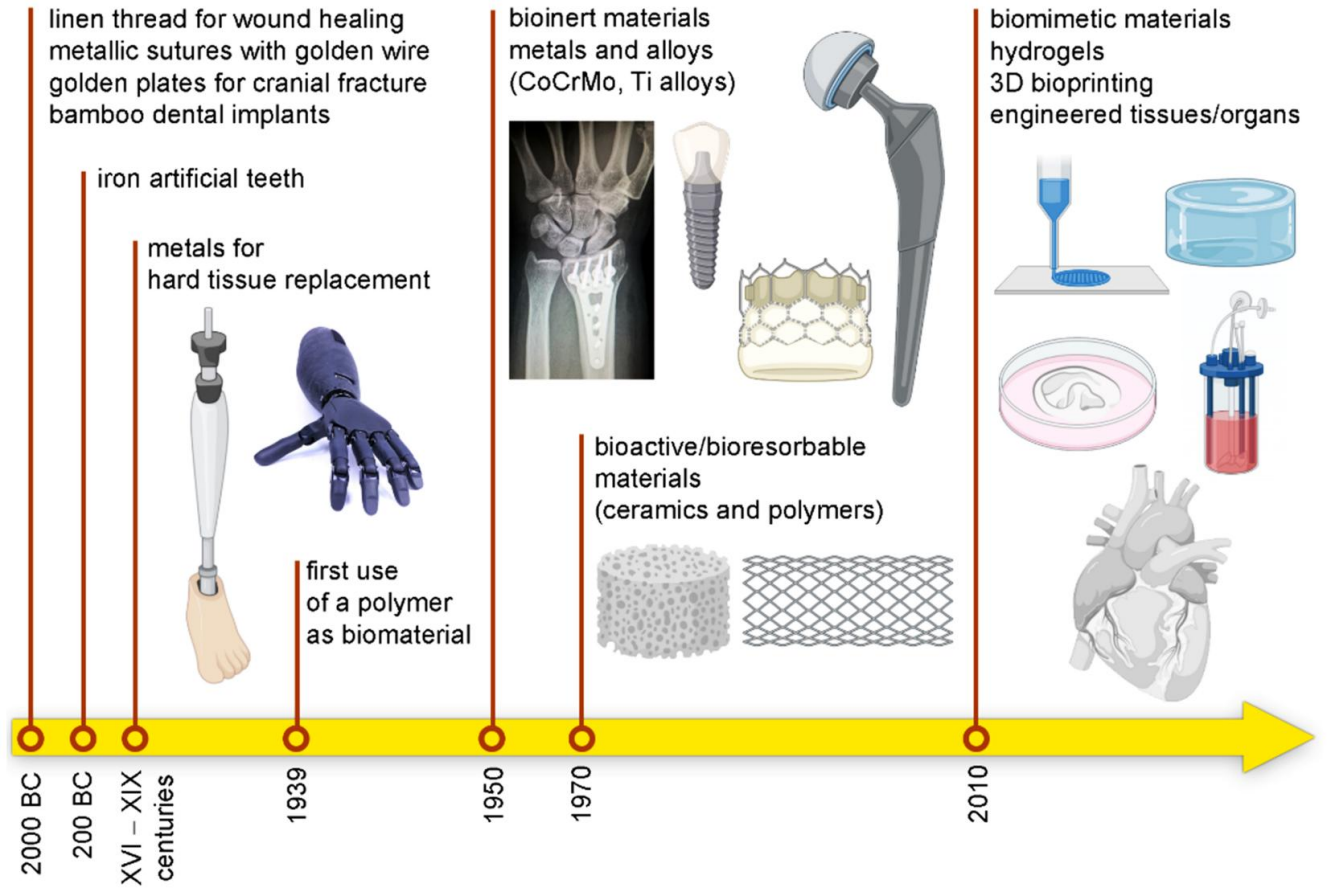
The development of biomaterials from bioinert stage to bioactive/biodegradable stage then to functional and smart stage. Obtained from Ref. [2]. Copyright ©2021, Todros *et al*.

Biomaterials are synthetic or treated natural materials and have been widely used to assess, rehabilitate, augment or supplement the physiological functions, tissues and organs within the human body [1]. In terms of evolution of biomaterials, it has experienced several evolutions or phases, including the initial exploratory phase, the subsequent bioinert phase, the bioactive/biodegradable phase and the current phase, which is characterized by the emergence of functional and intelligent biomaterials capable of eliciting precise responses at the cellular and molecular levels [2] (Figure 1). Within the domain of orthopedic applications, the utilization of biomaterials is circumscribed by stringent biosafety and efficacy imperatives, resulting in the selection and endorsement of mature biomaterials. Based on the statistical data, orthopedic biomaterials are predominantly utilized as components of medical devices and serve as integral elements in tissue engineering, specifically for the replacement and regeneration of tissues [3]. As the whole, orthopedic biomaterials are categorized into metals, ceramics, polymers and composites. Currently, biodegradable metallic materials especially magnesium (Mg) is garnering significant attention from researchers, while the obstacles to its orthopedic applications still exist.

Mg is one of the vital components of the human body and mainly stored in the musculoskeletal system as either a matrix component or an intracellular element for the homeostasis of various physiological functions. A series of physical and physiological properties of Mg has been investigated including mechanical adaptation, degradation, osteogenesis, angiogenesis, anti-bacteria, anti-inflammation and neuroprotection [4]. Mg has obtained substantial attention as a biodegradable material for medical implants attributed to its exceptional biocompatibility, which mitigates long-term toxicity and stress-shielding effects. However, its rapid degradation rate and inadequate mechanical performance in physiological environments pose significant challenges [5]. Though numerous companies have obtained regulatory approval for commercial application of their bioresorbable Mg-based orthopedic implants in regions such as Europe, South Korea and China, Mg-based orthopedic implants are still rarely used clinically. The first Mg-based orthopedic implant in China developed by Eontec Co., Ltd (China) has received CE approval and is in the stage of product registration. This review aims to explore the current challenges and future directions of Mg-based medical implants for clinical translation. To discover the application prospect of Mg, this article provides an update on biomaterials used in orthopedics and analyses the factors driving orthopedic innovation. The translational pathway of Mg is systematically described to provide a reference frame for the translation of novel materials and promote the development of innovative orthopedic biomaterials for clinical applications.

The physiology of magnesium ions (Mg^2+^) and its potential clinical applications are reviewed as the basis of translation. In order to realize the orthopedic application, modification of the performance of Mg as implantable metals and function of the degradation products of Mg *in vivo* are described. For the clinical needs of treating the steroid-associated osteonecrosis (SAON), Mg screws and Mg-based composite porous scaffolds (Mg/PLGA/TCP: magnesium/poly(lactic-co-glycolic acid) (PLGA)/tricalcium phosphate (TCP)) have been developed, but the impediments associated with the utilization of Mg-based implants in weight-bearing skeletal sites persist. To utilize the beneficial biological effects of Mg degradation and overcome the weakness in mechanical stability for fracture fixation, the concept of developing Mg/titanium (Ti) hybrid orthopedic implants is reported, where the Ti component provides effective mechanical support while the inclusion of Mg component potentially optimizes the biomechanical properties of Ti component and facilitates bone healing. Finally, the translation process of Mg-based medical devices is stated to promote its clinical translation as innovative medical implants. With the increase of beside needs and the maturity of novel technology, such as 3D printing and nanomaterials, the Mg as an emerging bioactive material will have more diversified and promising orthopedic applications.

## Orthopedic biomaterials

### Metals

**Figure 2.**
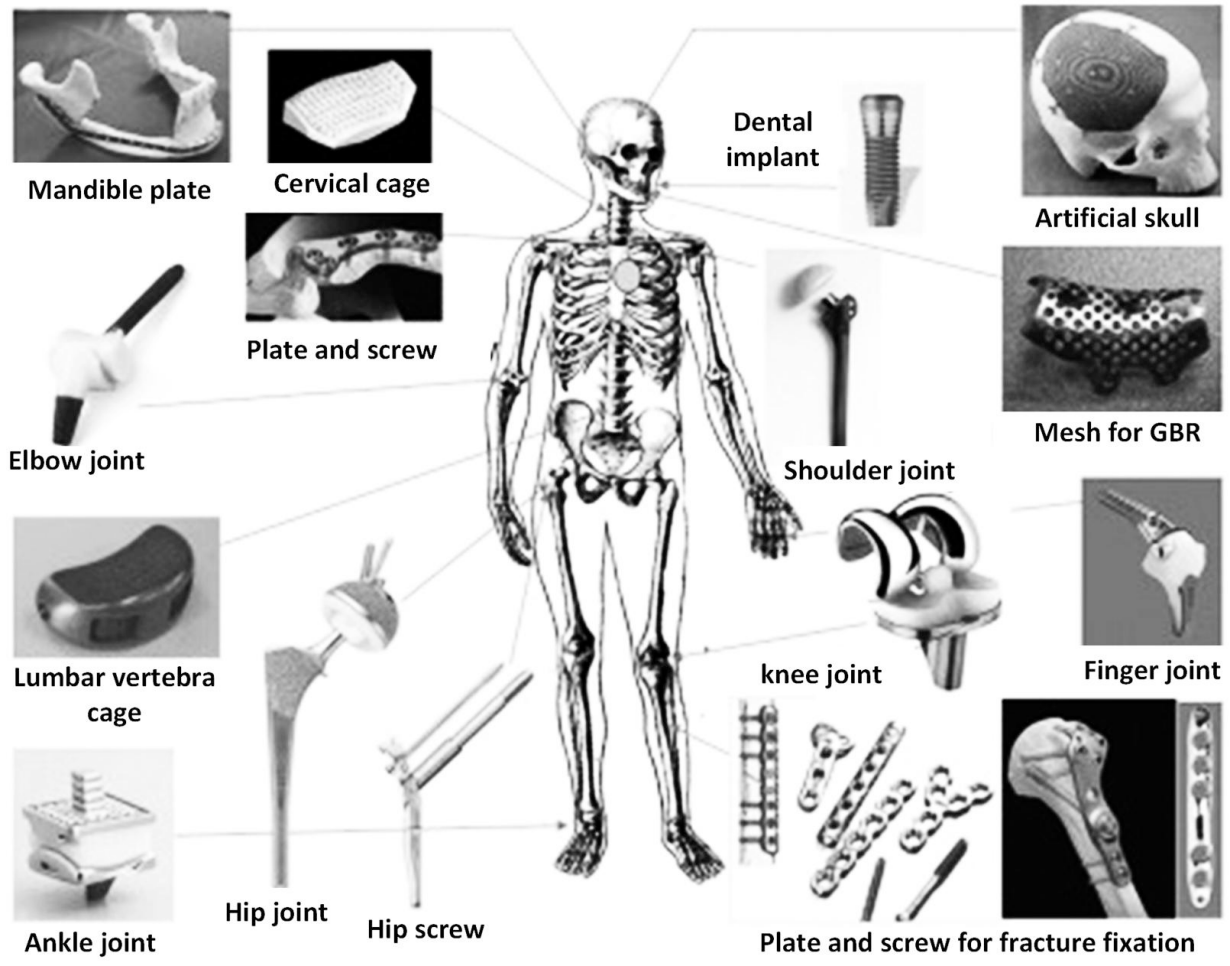
Orthopedic biomaterials used as medical devices. Reproduced from Ref. [3] with permission of Elsevier ©2019.

The metal and metal alloys have advantages being biocompatible, high strength with reasonable corrosion resistance and have been extensively employed within the orthopedic domain as load-bearing components, including stainless steel, cobalt–chromium alloys, tantalum, Ti and Ti-based alloys. However, the poor wear resistance limits its application in the articular surfaces of prosthetic devices and the generation of wear particle at the implant interface increases the risk of ionic toxicity [6]. In addition, the high Young’s modulus of metal and its alloys may produce the stress shielding of the surrounding bone leading to the occurrence of bone resorption, which further affects the integrity of bone regeneration and the osseointegration stability of orthopedic implants. Mg and its alloys, as emerging biodegradable metals, exhibit great potential for orthopedic application due to crosslink with host tissue as the degradation and will be introduced in the next context. Figure 2 shows the orthopedic biomaterials used as medical devices.

### Ceramics

Ceramics are classified as bioinert and bioactive based on their interaction with biological systems. Bioinert ceramics exemplified by alumina (Al_2_O_3_) and zirconia (ZrO_2_) have superior wear resistance and high toughness to render them suitable for applications as femoral head components, liners, artificial knee and dental implants. Bioactive ceramics are used to repair damaged bone, fill the void after resection of bone tumors, repair and fusion of vertebrae or as the coating to promote osteogenesis. Hydroxyapatite and calcium phosphates are well-admitted ceramics as bone substitutes and coating, which could release ions to stimulate osteogenesis in the physiologic fluids with the extension of the implant period [7]. Bioactive glass, such as bioglass 45S5, is proposed to distinguish from bioinert materials and expand to silicon glass, calcium glass, etc. The performance of bioactive glass is diversified with the compositional changes to promote osteogenesis, angiogenesis, anti-inflammatory and antibacterial, while the challenge is low tensile strength and toughness [8]. To increase the toughness of glass, glass-ceramics are synthesized by controlling the crystallization of glass [9]. Due to the satisfactory biocompatibility and compression resistance, Li_2_O–Al_2_O_3_–SiO_2_ glass-ceramics are fabricated by digital light processing to form the porous structure to promote osseointegration and withstand physiological loads [10].

### Polymers

Polymers have garnered extensive application in orthopedic load-bearing skeletal sites with high strength, toughness, wear resistance or Young’s modulus close to the bone, including carbon fiber, ultra-high molecular weight polyethylene (UHMWPE), PEEK and polymethyl methacrylate (PMMA). Carbon fibers have been described for use in internal fixation for orthopedic surgeries, but the potential release of carbon debris into the surrounding medium may cause systematic toxicity and soft tissue-related complications [11]. UHMWPE is a polymer of polyethylene with a molecular weight ranging from 2 to 6 million, which has been used for acetabular liner in total hip replacement and total knee replacement and introduced by Charnley in 1962, marking a significant milestone in orthopedic surgery [12]. In a comparative wear analysis between UHMWPE and highly cross-linked polyethylene (XLPE) with the same simulator and methods according to ISO 14242, the wear rate of the XLPE is lower than UHMWPE [13]. The PEEK has been employed for skull repair, interference screw and intervertebral fusion owing to its modifiability, bone-like modulus and capacity to mitigate stress shielding. Polymethyl methacrylate (PMMA) is recognized as a bone cement used for implant fixation. The composition of PMMA includes PMMA copolymer, barium oxide (as a radiopaque agent) and benzoyl peroxide (as a polymerization initiator) [14]. The aforementioned polymers are bioinert, while the biodegradable polymers composed of natural biodegradable polymers such as chitosan, hyaluronic acid, gelatin, collagen, silk, alginate and cellulose, and synthetic biodegradable polymers such as polyglycolic acid, polylactic acid and copolymers [15]. Hydrogel is a macromolecular material with high hydrophilicity and can be made of scaffolds with osteogenesis and angiogenesis or complexed with various materials or drugs to achieve the desired therapeutic performance [16]. Due to the rapid degradation rate and alkaline environment of Mg-based materials, combining with the polymers to modify the physical performance and biocompatibility of Mg-based materials is a promising direction for further research and clinical translation.

### Functional composites

Functional composites leverage the responsiveness to exogenous and/or endogenous stimuli to achieve controlled therapeutic effects, including antibacterial, antitumor, osteogenic and angiogenic properties [7]. Single or multiple physical signals including photothermal, mechanical, acoustic, electrical and magnetic signals are used to induce the response of functional composites. Based on the photothermal effect, when the black phosphorus (BP)-tagged responsive strontium (Sr) hydrogel particles are exposed to near-infrared irradiation, the microparticles experience volume shrinkage and release encapsulated Sr ions, to attain the therapeutic objectives of anti-inflammation, anti-apoptosis, bacterial inhibition and osteogenic stimulation [17]. Mechanical responsive piezoelectric PLLA nanofiber scaffolds promote cartilage generation and regeneration in a rabbit model under conditions of mechanical stress or articular loading [18]. Ultrasound-responsive PLLA/Au@Potassium niobate scaffold with sonodynamic therapy is used to solve the implant-related infection [19]. Magnetic-responsive hydrogel incorporated magnetic iron oxide nanoparticles demonstrate responsiveness to alternating magnetic fields, which possess the capacity for profound tissue penetration to realize osteogenic differentiation, angiogenic differentiation and biomineralization processes within pre-osteoblastic cells [20]. Despite significant progress in the field of functional composites, considerable challenges remain before achieving successful clinical application. It is essential to analyse the factors driving innovation in order to facilitate clinical translation. Table 1 shows the common biomaterials for orthopedic implantable devices.

**Table 1.** The common biomaterials for orthopedic implantable devices [15, 21]

Biomaterials	Application for implantable devices	Advantages	Disadvantages
Stainless steel	Wires, pins, screw–plate system, screw–rods system and intramedullary nails	Biocompatible, high strength and corrosion resistance	Poor wear resistance
Cobalt–chromium alloys	Joint prosthesis	Biocompatible, high strength, corrosion resistance and wear resistance	Ionic toxicity
Porous tantalum	Joint prosthesis, interbody fusion cage, bone substitutes	Biocompatible, high strength, corrosion resistance, porosity, friction coefficient and low elastic modulus	High density and melting point
Ti and Ti-based alloys	Wires, pins, screw-plate system, screw-rods system, intramedullary nails, interbody fusion cage and joint prosthesis	Biocompatible, high strength and corrosion resistance	Poor wear resistance
Al_2_O_3_ and ZrO_2_	Liner of joint prosthesis	Biocompatible, high compression strength, corrosion resistance and wear resistance	Poor bending strength and fracture toughness
Bioglass 45S5	Bone substitutes	Biocompatible, degradable, high bioactivity, angiogenic osteogenic and antibacterial properties	Brittleness
Calcium phosphates	Bone substitutes	Biocompatible, degradable, high bioactivity, osteogenic properties	Brittleness
Carbon fiber	Screw–plate system, interbody fusion cage	Biocompatible, high strength-to-weight ratio, durability, elastic modulus close to bone	Bioinert
Polyethylene (UHMWPE, XLPE, etc)	Liner of Joint prosthesis	Biocompatible, high strength, wear resistance and impact resistance,	Oxidative degradation, wear particle
PEEK	Screws, interference screw, screw-rods system, interbody fusion cage	Biocompatible, high strength, wear resistance and elastic modulus close to bone	Bioinert
PMMA	Fixation of prosthesis and filling the defect bone	Biocompatible, high strength, durability	High polymerization temperature and polymer monomer cytotoxicity
PLA and PLA-based polymers	Screws, interference screw and bone substitutes	Biocompatible, degradable, osteogenic and antibacterial properties	Low strength and uncontrolled degradation

## Driving force for innovation of orthopedic implants

### Technology-driven innovation

With the development of cutting-edge technology, 3D printing, surgical robots, artificial intelligence and machine learning have been introduced and applied in orthopedics. Amongst them, 3D printing with the convenience of personalized medicine and precise medicine expands the scope of biomaterials applications. Ti alloys have been extensively employed in orthopedics while its Young’s modulus is higher than bone causing the subsidence or loosening of implants in load-bearing skeletal position. Optimizing the implant structure to align the mechanical performance of Ti alloys with surrounding bone tissue constitutes a strategic approach. To mitigate the occurrence of bone resorption and implant loosening, porous lattice structure and performance evaluation were proposed as viable solutions [22]. Furthermore, if the porous structure with negative Posson ratio is integrated into implants, it will not only provide the porous space to promote bone ingrowth but also stimulate the surrounding bone in compression avoiding the bone resorption induced by stress shielding [23]. However, the Young’s modulus of Mg is close to bone and reduces the risk of stress shielding. The 3D printing brings the opportunity to manufacture specific implants and achieve controllable degradation of Mg [24]. Liu *et al.* [25] fabricated porous anatomical scaffolds composed of WE43 (Mg alloy containing 4 wt% yttrium and 3 wt% rare earth elements) and treated the scaffold with high temperature oxidation to slow down the degradation rate. Following implantation into bone defects, the scaffolds' porous structures were maintained for over 12 weeks in the absence of penetration and for more than 6 weeks in the presence of penetration. Yet, the biological effects for enhancing bone regeneration contributed by Mg degradation could be limited at the early phase after implantation. Similar to 3D printing, bio-fabrication is capable of precisely imitating the structure and function of host tissue to form bio-engineered scaffolds by assembling biomaterials and cellular components. The bioink includes the nanomaterials, cells, drugs and growth factors that could endow the Mg-based scaffolds with the requisite properties to meet specific biomedical objectives [26]. The more closely the scaffold mimics the native tissue, the more effectively it can rehabilitate the damage. These aspects include biomechanical properties like mechanical strength and structural architecture, chemical behavior such as the regulation of cytokine expression, and cellular response modulation, which encompasses the recruitment and differentiation of cells, may still be the main scope of Mg-based scaffolds fabricated by 3D bioprinting [26].

### Market-driven innovation

To reduce the financial burden on healthcare systems, volume-based procurement strategy, which aims to decrease the price of products by volume purchase, is implemented in China and accounts for over 80% of the orthopedic implant market. In another aspect, the National Medical Products Administration (NMPA) supports the research and development (R&D) of innovative medical devices to promote the product evolution and thereby enhance therapeutic effect. Innovative medical devices are exempt from volume-based procurement thereby allowing them to seize more market share and seek additional profit margins. At present, artificial bone substitutes and 3D printed products have not been incorporated into the realm of volume-based procurement and represent the translation direction of orthopedic materials. Mg as an emerging bioactive material is still in the infancy stage for replacing or partially replacing the conventional orthopedic implants currently used in clinics. However, the market-driven innovative implants may not always be superior to previous ones. New generations of knee prosthesis aims to increase the patellofemoral conformity compared with previous version, while the posteriorly positioned stem escalates the risk of posterior tibial cortex impingement, which may precipitate subsequent discomfort and the incidence of periprosthetic fractures [27]. Critical opinion is desirable for market-driven innovative orthopedic materials to control clinical risks and enhance treatment efficacy.

### Clinical needs-driven innovation

Clinical needs are a significant source of implant innovation and bridge the gap between discovery, invention and clinical translation. The bio-design process is a highly-praised needs-driven methodology for innovation that originates in clinical discovery and is bolstered by the practical insights of surgeons and clinicians. With the rapid increase in population aging, the amounts of surgical operations in orthopedics are expected to increase. For clinical needs-driven innovation, the application of the artificial hip joint to replace end-stage osteoarthritis was a pioneering work [12]. To achieve the fixation of the stem with surrounding bone, bone cement made of PMMA is employed, adhering to the principles of force-closure and shape-closure [14]. However, the poor cement technique, augmented stem offset, reduced stem length, coarse surface finish and circular cross-sectional stem design are all factors that have been exposed to cause the failure of cemented stem [28]. The non-cemented stem, serving as an alternative, is used to provide the primary stability through osseointegration with the implant. To enhance osteoconduction and facilitate bone apposition onto the implant surface, sufficient primary stability, suitable surface topography and biocompatible prosthetic material are all required [21]. According to previous studies, uncemented fixation was associated with a significant increase in the risk of aseptic revision for the management of hip fractures via hemiarthroplasty, as compared to cemented fixation methods [29]. The clinical needs-driven innovation promotes the resolution of clinical challenges and enhances the optimization of therapeutic strategies to achieve superior outcomes. In the most promising application area of Mg as fixation screw and/or plate in orthopedics, the physical properties expected to meet bedside needs remain to be investigated. It is known that the load exerted on the hip joint during a gait cycle is approximately 2.5–3 times of the individual's body weight [22]. Mg-based implants in weight-bearing parts of lower limb need to be sufficiently strong to support this load and possess adequate fatigue resistance to endure the repetitive stress of daily activities in patients with hip fractures. In addition, Mg-based implants are generally expected to be subjected to 150 000–250 000 cyclic loads in the first 2–3 postoperative months [30].

Consequently, Mg-based biomaterials for orthopedic applications should regard clinical issues as the initial driving factor and introduce advanced engineering technology and scientific theory to address these issues and validate their efficacy in clinical. During this process, the market factor promotes the extension of Mg-based orthopedic biomaterials. As the pivotal step in aligning clinical indications with Mg-based biomaterials, it is necessary to link up with biological and pathophysiological conditions for developing Mg-based biomaterials for clinical applications.

## Bone biology- and pathophysiology-based metallic screw-plate system design in osteoporotic fracture

### Biology and pathophysiology of osteoporotic fracture

**Figure 3.**
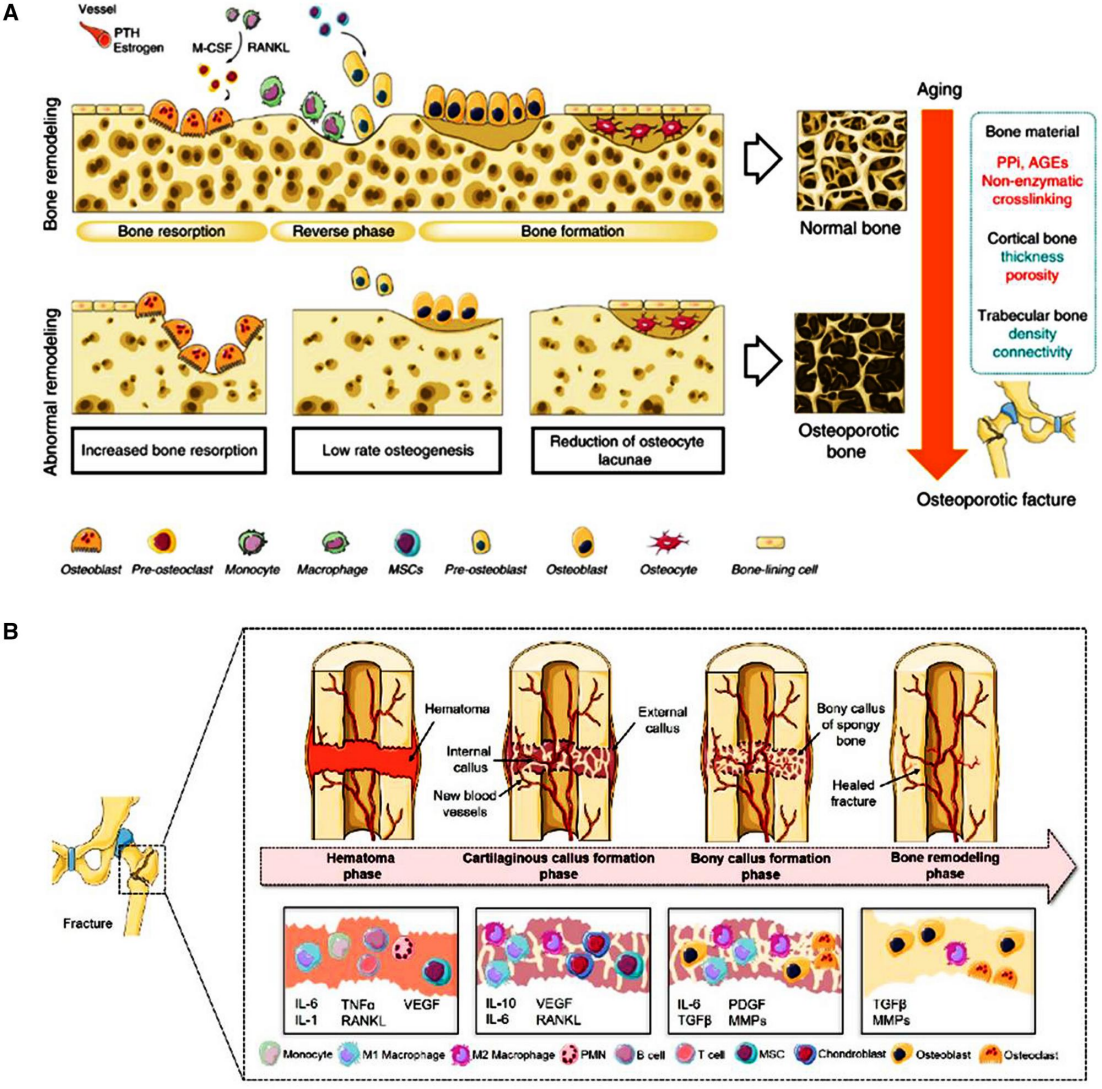
Bone remodeling of osteoporotic bone and the process of bone healing. (**A**) Bone remodeling in osteoporosis. Obtained from Ref. [31]. Copyright ©2019, Xie *et al*. (**B**) Bone healing process. Obtained from Ref. [33]. Copyright ©2021, Pfeiffenberger *et al*.

Accompanied by aging, osteoporosis, a prevalent systemic skeletal disorder, is increased and hallmarked by reduced bone mass and compromised bone microstructure [31]. Osteoporosis increases the risk of osteoporotic fractures, commonly in the spine, hip and forearm. The complex interplay of biological and pathophysiological mechanisms impedes the normal healing process of osteoporotic fracture and poses a significant challenge in clinical management. Within the scope of bone remodeling of osteoporotic, an imbalance between osteoclast and osteoblast activity results in a negative bone mineral balance, accompanied by the senescence of osteocytes and ultimately culminates in reduced bone formation and regeneration (Figure 3A) [32]. In addition, non-pathological fracture healing entails a well-coordinated regenerative process aiming to restore the bone's original structure and cellular composition. This post-fracture healing process involves hematoma formation, granulation tissue formation, bony callus formation and bone remodeling, orchestrated by various cell types including immune cells, bone-forming cells, chondrocytes, osteoclasts, vascular cells, etc [31].

During osteoporotic fracture healing, the pathophysiological milieu undergoes significant alterations. Chronic low-grade inflammation induced by cytokine accumulation, such as IL-6, TNF-α and IL-1β, creates a pro-inflammatory environment that promotes osteoclast differentiation and inhibits osteoblast formation (Figure 3B) [33]. Simultaneously, oxidative stress exacerbates bone loss through increased intracellular reactive oxygen species (ROS) production and activation of detrimental pathways [34]. The interaction between the immune and skeletal systems is crucial, notably in osteoporotic fracture scenarios where the immune cell functions are impaired and interactions are disrupted. Estrogen deficiency, a primary cause of osteoporosis, stimulates T/B cell expansion and osteoclastogenic factor production which leads to the overactivation of the osteoclasts [35]. Moreover, in osteoporosis, mesenchymal stem cell lineage commitment favors adipocyte differentiation over osteoblasts, originating from the same progenitor pools. This skewed lineage commitment leads to bone marrow adipose tissue accumulation and diminished osteoblast populations [31]. Additional factors such as leptin and epigenetic regulation exert significant influence on bone metabolism, promoting adipogenesis while inhibiting osteogenesis in bone marrow MSCs [36]. Furthermore, the complex relationship between the immune system, central nervous system, gut microbiome and bone turnover regulation is increasingly acknowledged with aging [37].

In brief, the distinct osteoporotic environment compromises bone regenerative capacity, resulting in delayed initial healing, prolonged recovery times and a challenging prognosis for osteoporotic fracture repair. These multifaceted interactions underscore the need for targeted interventions and a comprehensive understanding to address the complexities inherent in osteoporotic fracture healing dynamics.

### Design principle of metallic screw–plate systems

**Figure 4.**
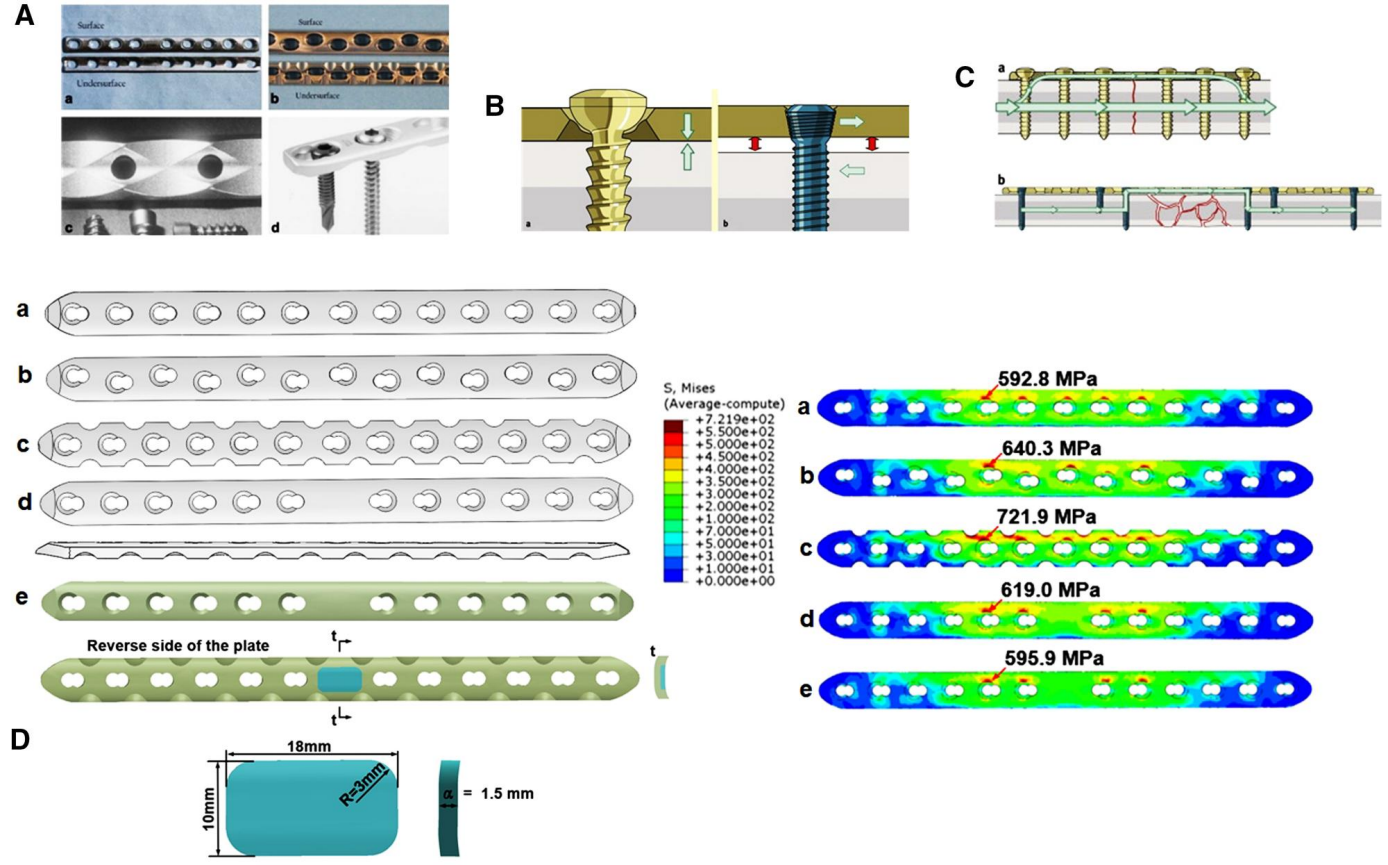
Design principle of metallic screw–plate systems and modification of the common screw–plate systems to reduce stress shielding. (**A**) The metallic screw–plate system. Reproduced from Ref. [39] with permission of Elsevier ©2007. (**a**) Dynamic compression screw–plate (DCP) system, (**b**) limited contact dynamic compression screw screw–plate system (LC-DCP), (**c**) point contact screw–plate system, (**d**) locking compression screw–plate (LCP) system. (**B**) Fixation with DCP and LCP. Reproduced from Ref. [42] with permission of Elsevier ©2009. (**C**) Transfer of loads in DCP and LCP. Reproduced from Ref. [42], with permission of Elsevier ©2009. (**D**) Common metallic screw–plate systems and stress distribution under the compression load of 1500 N. Adapted from Ref. [43] with permission of Elsevier ©2024. (**a**) Traditional plate, (**b**) asymmetric plate, (**c**) stress dispersed plate, (**d**) central enhancement plate, (**e**) modified plate.

Fixation principles of bone fracture include Arbeitsgemeinschaft für Osteosythese (AO) and biological osteosynthesis (BO). Correspondingly, design principle of the metallic screw–plate system can be divided into three types: dynamic compression screw–plate (DCP) system, limited contact dynamic compression screw–plate (LC-DCP) system and locking compression screw–plate (LCP) system (Figure 4A). The DCP system is designed to fix the fractured bone as stable as possible without considering the effect of micromotion on fracture healing. Although the DCP system provides stable internal fixation and a low incidence of delay union and nonunion, it has obvious limitations, including a slow healing process, subcortical bone loss leading to osteoporosis and the potential risk of stress concentration and fatigue cracking [38]. Therefore, the LC-DCP system is further developed based on the evidence that excessive contact between the cortical bone and the screw–plate system could cause vascular occlusion, which in turn could lead to cortical bone necrosis [39]. However, the LC-DCP system does not have significant advantages in fracture healing compared to the DCP system [40]. Furthermore, a point contact screw–plate system has been developed based on the LC-DCP system in which the contact area between the cortical bone and the screw–plate system is reduced to point contact. The benefits of the point contact screw–plate system include a faster healing process, simpler surgical procedures and fewer surgical complications [41]. However, one of the clinical challenges that remains to be addressed is the issue of osteoporosis beneath the plates after long-term implantation. As a result, the concept of the LCP system was developed based on the evidence that micromotion at the fracture site could promote fracture healing and reduce the incidence of delayed union and nonunion (Figure 4B and C) [42]. The LCP system could accelerate fracture healing, promote callus formation and provide superior biomechanical properties of the healed tissue and attributed to the biological fixation mechanism, which allows for controllable micromotion, minimal implant-to-bone contact, extended-span bridging and a reduced number of screws required for stabilization [38]. The common LCP system used in clinic can be classified as traditional plate, asymmetric plate, stress dispersed plate and central enhancement plate according to the distribution of the holes and features of plates [43]. The stress distribution of these plates under the load of 1500 N is shown in Figure 4D. To decrease the maximum stress and homogenize the stress distribution, the materials with the modulus close to bone are introduced to plate (Figure 4D).

### Challenge of metallic screw–plate systems

The LCP system has the potential to significantly promote fracture healing, while several clinical challenges of metallic screw–plate systems remain to be addressed. First, most metallic screw–plate systems, primarily composed of stainless steel, Ti and Ti alloys, remain in the body unless a secondary surgical procedure is needed for their removal. This may potentially lead to adverse effects, including inflammation or allergic reactions resulting from wear, impingement and corrosion of screws and plates. Second, the metallic screw–plate system in load-bearing skeletal position can be prone to fatigue fracture after implantation if nonunion occurs and may need secondary surgical procedures depending on the clinical conditions [30]. Third, the mismatch of Young’s modulus between the bone and the metallic screw–plate system could cause stress shielding. Fourth, the application of the metallic screw–plate system for complex fractures, such as osteoporotic fractures and comminuted fractures, still carries a higher risk for delay union or nonunion [43]. The advantages of Mg provide a potential strategy for these challenges.

## Mg as biodegradable and bioactive metals for orthopedic applications

### Physiology of Mg ions and application prospect

Mg^2+^ serves as an essential co-enzyme of over 200 proteins to modulate human physiology. Numerous advantages of Mg have been discovered including maintaining bone formation, modifying muscle function, promoting cardiovascular health, protecting nerves and treating constipation and diabetes. Reports have indicated Mg^2+^ deficiency in osteoporotic patients [44]. For the direct effect, Mg^2+^ deficiency leads to large hydroxyapatite crystals and decreases bone stiffness. Meanwhile, the number of osteoblasts decreases and the number of osteoclasts increases due to lack of Mg^2+^. For indirect effect, the decrease of parathyroid hormone postpones the bone formation by decreasing the supplementation of vitamin D. There is no doubt that Mg is also an attractive material for orthopedic application because the mechanical properties of Mg are similar to nature bone. Though there are concerns on the toxicity of the Mg that excessive levels of Mg^2+^ can lead to hypermagnesemia, yet high serum level of Mg^2+^ is a cumulative process and the degradation process of Mg will release Mg^2+^ into the surrounding tissue. These ions are then metabolized and eventually excreted through the kidneys, the main organ in the body responsible for Mg^2+^ homeostasis. Clinical trials on biodegradable Mg-based materials have further indicated that degradation products of Mg-based implants could be tolerated in the body across the entire treatment periods [45, 46]. As the degradable metal, the *in vivo* implantation of Mg implant could avoid second surgery for its removal, which may reduce 30% of orthopedic surgeries, escape from stress shielding induced bone loss, reduce pain and decrease infection risk [47]. Mg-based implants exert multifaceted influences on osteogenesis, mediating direct and indirect effects on the interconnected bone, vascular, nervous and immune systems, thereby facilitating the potential for functional bone regeneration [4]. Further from the fundamental point, the Mg^2+^ ions released from Mg-based materials are transported into dorsal neurons via the mediation of Mg transporter 1 (MagT1) and transient receptor potential cation channel subfamily member 7 (TRPM7), thereby promoting the release of calcitonin gene-related peptide (CGRP). CGRP subsequently engages with its receptor, which is expressed on the surface of periosteal stem cells (PSCs), inducing the binding of cyclic adenosine monophosphate (cAMP) to its response element-binding protein. This results in the upregulation of osterix (SP7), which robustly stimulates osteogenesis, particularly in the periosteal region [48]. In addition, Mg-based nanomaterials exhibit superior tribological properties, high resistance to wear and tear, and the ability to sustain drug release [49]. For example, Zheng *et al.* developed engineered MgO nanoparticles to facilitate cartilage–bone synergistic therapy through the PI3K/AKT signaling pathway [50]. In fact, Lambotte used Mg to treat supracondylar humerus fractures and obtained the success in 1932, while Verbrugge failed to remodel the transdiaphyseal humerus fracture due to fast corrosion of Mg in 1937. From 1948 to 2010, there are no reports on clinical application of Mg-based orthopedic implants [51]. The rapid degradation of Mg with low purity causes the formation of gas and the earlier loss of mechanical support, thereby constraining the clinical application of Mg in orthopedics. Although Mg-based implants demonstrate promise in surgical treatment of musculoskeletal diseases, further R&D is necessary to enhance the corrosion resistance and mitigate the rapid degradation of Mg-based orthopedic devices before their clinical application. Table 2 shows the mechanical performance and corrosion resistance of potential Mg-based biomaterials in orthopedic applications.

**Table 2.** The mechanical performance and corrosion resistance of potential Mg-based biomaterials in orthopedic applications [4, 24]

Mg-based biomaterials	Microstructure and mechanical properties	Corrosion resistance
Pure Mg	Ultimate tensile strength of 100–140 MPa	≈30% volume loss after 24 weeks
AZ31	Hardness and elastic modulus values shown to be approximately 1.7- and 1.2-fold those of pure Mg, respectively	A fast corrosion rate and lack of stability of implants
AZ91	Formation of Mg_17_Al_12_ phase along the grain boundaries refined grain size and improved tensile strength	Accelerated corrosion rate compared to pure Mg due to the higher electrode potential of the Mg_17_Al_12_ phase
WE43	Enhanced ultimate tensile strength when adding <3% Y and <6% Nd. Improved compressive yield strength by the addition of 2, 4 and 6 wt% Y	Mg_12_Nd acted as corrosion barrier during the corrosion process
LAE442	Improved strength and refined microstructure with the addition of Nd and Ce at a concentration of 0.6% and 2%, respectively	Decreased corrosion resistance by the combination of Al and Nd
ZK60	Relatively high yield strength and high mechanical stability because of the Mg–Zn	Refined grain size, and purified the composition of the alloy, improved corrosion resistance due to the Zr element
Mg–Ca	Poor ductility due to the participation of Mg_2_Ca on grain boundaries and inside the grain for Ca content above 1.5 wt%	Exhibited acceleration of the corrosion rate due to high electrochemical activity of a large amount of beta phase (Mg_2_Ca) than α-Mg.
Mg–Zn	Improvement in the hardness, ultimate yield, tension, and compression with Zn content until 5%	Improved the corrosion resistance with increasing Zn content in the range of 1–5 wt%
Mg–Sr	Improved ultimate and yield tensile strength by increasing the amount of Sr from 0 to 2 wt % due to reducing the grain size	Improved corrosion resistance shown when increasing Sr up to 1–2 wt%
Mg–Zr–Sr	Improved the ductility, and smoothed grain boundaries achieved with the addition of between 1% and 5% Zr	Reducing the biocorrosion resistance considerably with the addition of excessive Zr and Sr to >2%
Mg–Zn–Ca	Increasing the Zn content resulted in a significant increase in the ultimate tensile strength	Ternary systems have the dominant mechanism for higher corrosion resistance

### Modification of the performance of Mg

To improve the performance of Mg implants, enhancing the purity of Mg, incorporating alloying elements into the Mg matrix and applying surface coatings on Mg are all potential approaches to decrease the degradation rate of Mg-based implants. Upon rigorous evaluation, the limited number of alloying elements is deemed appropriate for use, with exhibiting either ambiguous safety profiles or lacking comprehensive toxicological data [52]. Therefore, the ultra-pure Mg may be the perfect implant material. In fact, the first approved orthopedic implant was Mg alloy in 2014 and evaluated as half the pain, half the suffering, half the costs and half the time for bone healing compared with traditional implant. The analogous outcomes are elucidated in the comparative investigation between the degradable Mg-based screws and permanent Ti screws to treat mild hallux valgus deformities [53]. Mg-based screws have been reported to be used in the area of hallux valgus, osteochondrosis dissecans, distal tibia, distal fibula, carpal, metacarpal/bones, proximal humerus, distal femur and proximal tibia. Lam *et al.* [54] presented the first clinical cases of biodegradable Mg screws to treat radial head fractures and capitellum fractures. The screw is radiolucency to observe the early process of fracture healing and reduction of fixation. In the non-weight-bearing skeletal site, Mg-based screws promote the formation of calcification matrix at the bone–implant interface and accelerate the new bone regeneration to replace the degeneration of implant within 1 year of implantation [55]. To treat the medial malleolar fractures, Mg–Nd–Zn–Zr alloy coated with Ca–P is reported for implantation in a surgical site with closed injuries to provide stable fracture alignment and promote fracture healing without breakage of the Mg-based screws [45]. However, the mechanism behind the favorable clinical outcome still needs to be investigated.

### Biological function of the degradation products of Mg

The biological effects of Mg used in orthopedics are ascribed to its degradation products during Mg implantation *in vivo*, including Mg^2+^, hydrogen gas (H_2_) and local alkaline environment, which interact with the surrounding cells and tissue matrix and elicit biological responses [56]. Mg^2+^ generated from the degradation of implants can permeate the bone matrix toward the periosteum, which is innervated by dorsal root ganglion (DRG) sensory and stimulate the periosteum-derived stem cells (PDSCs) to differentiate along osteogenic pathways and form new bone [48]. In the process of tissue healing, hypoxia represents a transient phenomenon. The degradation of Mg is oxygen-dependent, potentially sustaining a relatively hypoxic microenvironment surrounding the implant. The presence of an acidic microenvironment is associated with destroyed bone architecture and high occurrence of osteonecrosis in the animal model, while the alkalotic microenvironment demonstrated a potential protective effect against osteonecrosis versus the acidic microenvironment [57]. H_2_ can selectively reduce the hydroxyl radical and the most cytotoxic ROS rather than the ROS possessed physiological function. Therefore, H_2_ can be posited as an efficacious antioxidant intervention owing to its ability to rapidly diffuse across membranes to reach and react with cytotoxic ROS to protect against oxidative damage [58]. In addition, Mg has demonstrated significant efficacy against methicillin-resistant *Staphylococcus aureus* (MRSA)-induced osteomyelitis and promote the formation of peri-implant bone tissue [59]. The rationale reason of this observation is that Mg-based scaffolds induce an elevation in pH, an increase in Mg^2+^ concentration and a rise in osmolality. Especially, the augmented pH is regarded as a principal factor in mitigating bacterial adhesion and biofilm formation [60].

### Practice of Mg in non-weight-bearing skeletal sites to treat SAON

Hyperlipemia causes the fatty liver and destabilization of endogenous plasma lipoproteins, which combine the disruption of marrow fat and the formation of fat embolism. Based on the fat embolism, the mechanical stage, chemical stage and thrombotic stage emerge in succession and lead to osteonecrosis [61]. Osteonecrosis is characterized by necrotic bone marrow adipose tissue and hematopoietic cells, ghost osteocytes and empty osteocyte lacunae within trabecular bone [62]. Core decompression, osteotomy and both vascularized and non-vascularized bone graft transplantation are the recognized procedures to preserve the femoral head with necrosis. The inserted bone graft may slip and displace postoperatively without the fixation. To solve this clinical problem, Zhao *et al.* [46] presented an innovative high-purity Mg screw for fixation of bone flap procured from ilium for the repair of the necrotic femoral head. With the degradation of Mg, the diameter of Mg screws reduced by approximately 25% at 12 months after surgery. The Harris hip score exhibited a significant improvement with the application of Mg screw fixation as opposed to the non-fixation of Mg screws. To further validate the angiogenesis of Mg screw used in the necrotic femoral head, Ti screw was introduced as the control group and illustrated the Mg screw improved the blood supply of the femoral head [63]. In addition, the Mg screw is also used to treat displaced femoral neck fractures and obtained satisfactory clinical outcomes with a low incidence of complications, such as avascular necrosis and nonunion after the average 16-month follow-up [64]. Behind the favorable outcomes, the primary and secondary phases of Mg degradation products exert a substantial influence on bone regeneration during the degradation of Mg-based implants, rather than solely the role of Mg^2+^ [65].

**Figure 5.**
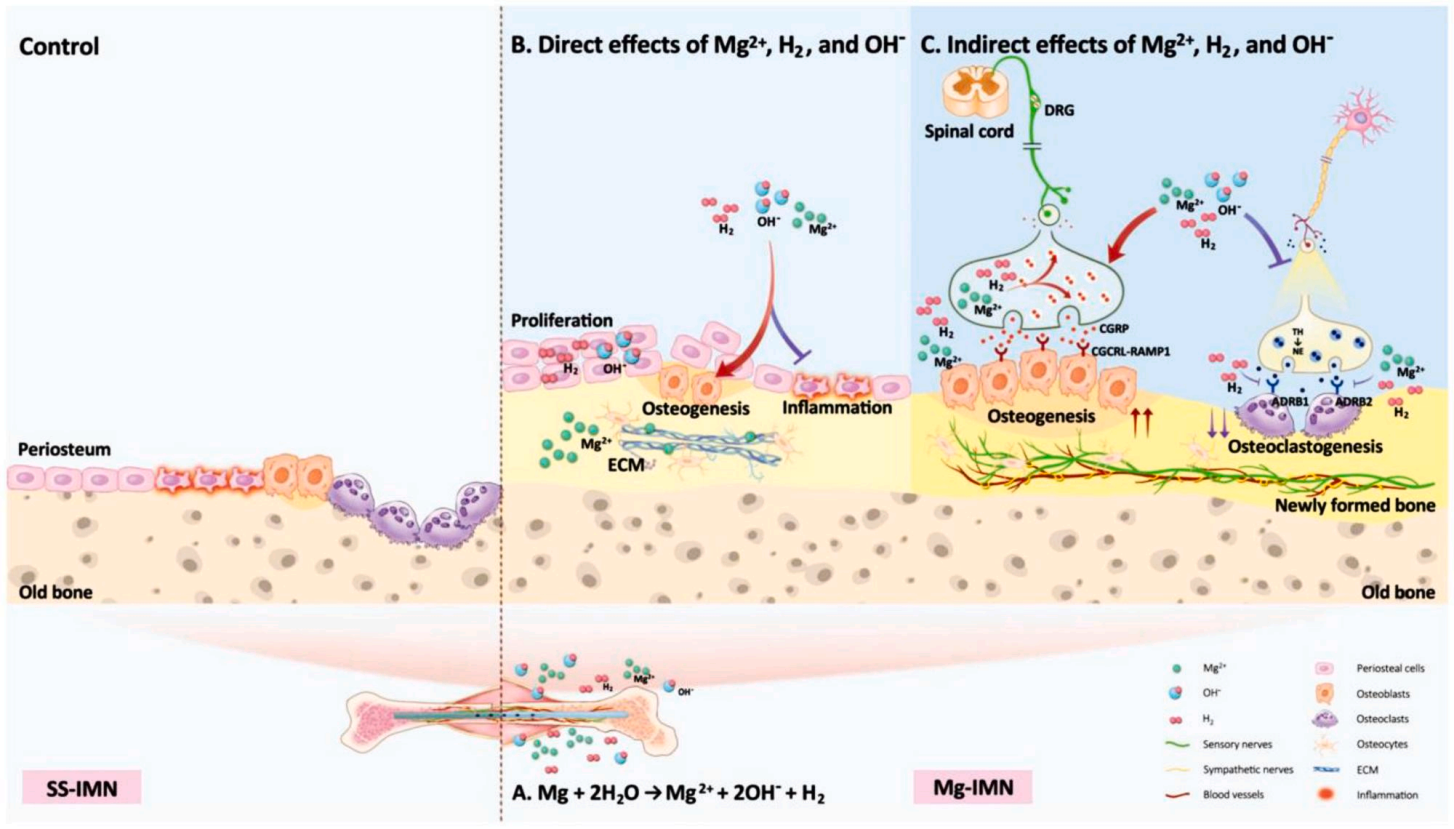
The function degradation products of Mg to regulate the osteoporotic fracture healing. Obtained from Ref. [66]. Copyright ©2025, An *et al*. (**A**) Degradation products of Mg. (**B**) Direct effects of Mg^2+^, H_2_ and OH^−^. (**C**) Indirect effects of Mg^2+^, H_2_ and OH^−^.

There are three distinct degradation products of Mg in fracture healing enhancement including Mg^2+^, OH^−^ and H_2_ [66]. In Figure 5, the Mg^2+^, OH^−^ and H_2_ could anti-inflammate and promote bone formation. Mg^2+^ and H_2_ upregulated the expression of CGRP to enhance osteogenesis. A degradable Mg screw developed in China had obtained approval for product registration multicenter clinical trial in 2019 and was certificated by CE in 2020. All cases were enrolled in the clinical study in 2023. Furthermore, the composite scaffold consisted of PLGA/TCP and icaritin is investigated as a bone defect filler subsequent to core decompression with the objective of preventing femoral head collapse in a bipedal SAON animal model using emu [67]. Based on these findings, the PLGA/TCP scaffold containing Mg is developed with a bio-mimic structure and enhanced mechanical characteristics [68]. Following implantation, PLGA/TCP/Mg scaffold demonstrates the capacity augment blood perfusion and facilitates neovascularization at the 4-week postoperative interval. Meanwhile, plenty of newly formed vessels with good architecture are observed at 8 weeks. Subsequently, at 12 weeks post-surgical intervention, PLGA/TCP/Mg scaffold significantly promote osteogenesis and enhance the mechanical integrity of the newly formed bone tissue.

### Challenges of the orthopedic application of Mg

**Figure 6.**
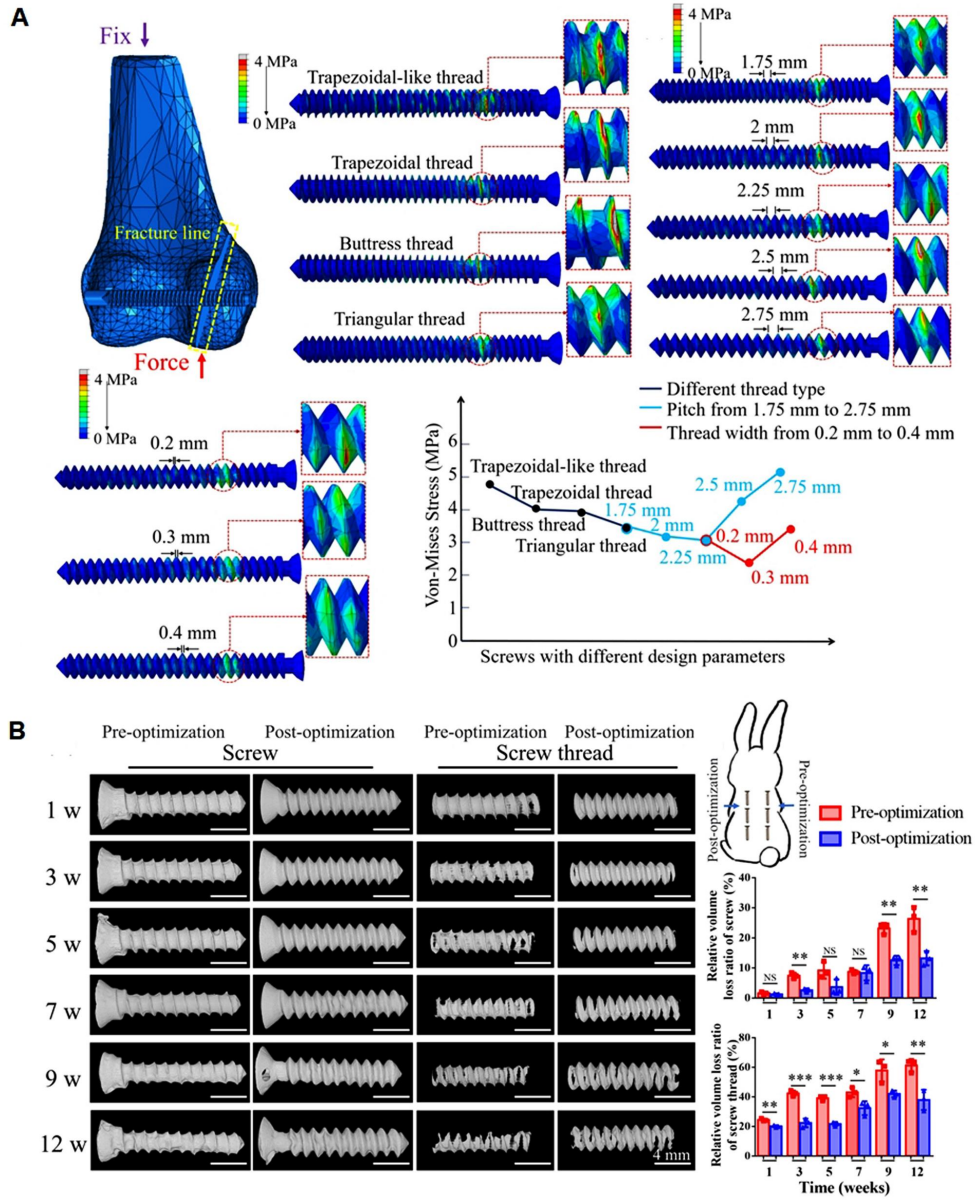
The optimized structure according to stress distribution decreases the degradation rate. Reproduced from Ref. [69] with permission of Elsevier ©2024. (**A**) Stress distribution of Mg screw with various features. (**B**) 3D morphology of the pre-optimized and post-optimized Mg screw inserted in the rabbit back subcutaneous tissue at different time period and statistics of loss ratio. NS: not significant, *P < 0.05, **P < 0.01, ***P < 0.001.

Compared with conventional inert materials, research on biodegradable metals is still in its nascent stage offering vast opportunities for innovation. Mg-based implants are provisionally required to maintain mechanical stability during the healing process of the injured tissues. Under stress corrosion conditions, there exist two principal mechanisms responsible for the initiation and propagation of cracks in Mg-based implants [70]. (1) Predominantly governed by the anodic dissolution mechanism, the oxide film on the surface of the alloy will be broken under the condition of extension, so that the Mg alloy matrix is exposed, partially dissolved and then cracks appear. Due to the sustaining action of the stress, cracks continue to initiate and develop and finally leading to the failure of Mg-based implants. (2) During the cathodic reaction of Mg-based implants, hydrogen atoms are generated, which infiltrate and diffuse through the Mg matrix. When the concentration of hydrogen reaches a certain value, it will cause cracks. The stress corrosion of Mg-based implants *in vitro* is affected by the surrounding environment and geometric design of Mg-based implants. Luo *et al.* calculated the stress distribution of Mg screws with different thread types, thread pitch and thread width using finite element analysis and optimized the structure of screws to retard stress corrosion in Mg-based implants (Figure 6) [69].

However, in load-bearing skeletal sites, especially in cases of bone fracture, the fracture gap is easy to generate micromotion influenced by local mechanical loading at the early stage of bone healing. Mg-based orthopedic fixators must possess robust initial mechanical integrity to provide secure support for the fractured bone at healing stage including inflammatory phase and soft callus phase. The degradation of the Mg-based implants may decrease their mechanical strength, so the degradation process of the Mg-based implants should be optimized to match the healing process of the fractured bone. However, the challenge still exists that biodegradable and bioactive metals as exogenous growth factors are expected early degradation while stable fixation implants are wished to slower degradation.

## R&D of hybrid orthopedic implants for fracture fixation and healing enhancement

### Conception of hybrid orthopedic implants

The compromised bone quality and impaired healing capabilities of osteoporotic fractures present a substantial challenge for orthopedic surgeons. Osteoporotic bone may not be suitable for using conventional rigid implants for fracture fixation due to their decreased bone mass and deteriorated bone structure, which can result in stress concentration and fracture fixation instability. Delays in callus formation and ossification have been reported, with failure rates of fixation in osteoporotic patients ranging from 10% to 25% [71]. Innovative solutions are necessary to improve the clinical application of Mg-based implants to alter delay union or nonunion, as there are concerns about their rapid degradation at load-bearing skeletal sites [56].

Numerous Ti/Mg hybrid fixation systems that include bioactive Mg implants with conventional non-degradable Ti internal fixation have been developed to meet the challenge in osteoporotic fracture fixation and healing enhancement (Table 3). The design of the Ti/Mg hybrid fixation system ensures that Ti implants provide sufficient mechanical strength while Mg implants facilitate fracture healing. Given that Mg possesses Young’s modulus and yield strength comparable to human cortical bone, its incorporation into the fixation system reduces the rigidity commonly associated with conventional implants. Furthermore, Mg has demonstrated the ability to stimulate the periosteum, enhancing callus tissue generation through the upregulation of CGRP, a protein known to promote fracture healing [48]. Galvanic corrosion of Mg and Ti may lead to rapid degradation of Mg due to the difference of electrode potentials (Mg: −2.356 V, Ti: −0.163 V). However, the corrosion rate of Mg in the Mg–Ti hybrid system is also influenced by the contact area ratio and surrounding medium. In order to prevent Mg corrosion, Fazel *et al.* [72] treated Mg–0.6Ca using plasma electrolytic oxidation to form a coating of hybrid couples to significantly decreased the strong galvanic degradation of Ti/Mg hybrid systems. As such approach may prevent the Mg degradation during early healing phase or tissue regeneration phase, we developed the Ti/Mg hybrid system without using Mg for bone fixation rather for early degradation even with wished underlying mechanism of galvanic corrosion. In fact, animal models published in our previous studies using Ti/Mg hybrid fixation systems showed rather faster degradation of Mg in favor of acceleration of fracture healing as our concept is to utilize Mg biological anabolic effects rather the mechanical properties where Mg compartment does not provide fracture fixation function [66]. Further application of these hybrid fixation systems to clinical trials should be supported by valuable insights and evidence.

**Table 3.** Summary of representative animal and simulation studies on Ti/Mg hybrid fixation systems as potential orthopedic implants

Hybrid system	Control	Surgery	Method	Major findings	Clinical significance	Ref.
Mg-containing Ti intramedullary nail system	Stainless steel	Femoral fracture	Animal study: rat	Accelerated fracture healing	Innovative approach to fracture fixation in heavy weight-bearing sites	[48]
Mg/Ti screw and plate fixation system	Ti alloy	Tibial fracture	Animal study: rabbit	Increased callous formation at fracture gap	Innovative approach to fracture fixation in heavy weight-bearing sites	[73]
Mg/Ti screw and plate fixation system	Ti alloy	Femoral fracture	Simulation study: finite element analysis	Improved mechanical properties while exerting biological effects	Innovative approach to fracture fixation in heavy weight-bearing sites	[74]
Mg–Ti hybrid cannulated screws	Ti alloy	Femoral neck fracture	Simulation study: finite element analysis	Improved mechanical properties while exerting biological effects	Innovative approach to fracture fixation in heavy weight-bearing sites	[75]
Mg–Ti hybrid plate fixation system	Ti alloy	Femoral fracture	Simulation study: finite element analysis	Improved mechanical properties while exerting biological effects	Innovative approach to fracture fixation in heavy weight-bearing sites	[43]

### Clinical translation process of hybrid orthopedic implants

**Figure 7.**
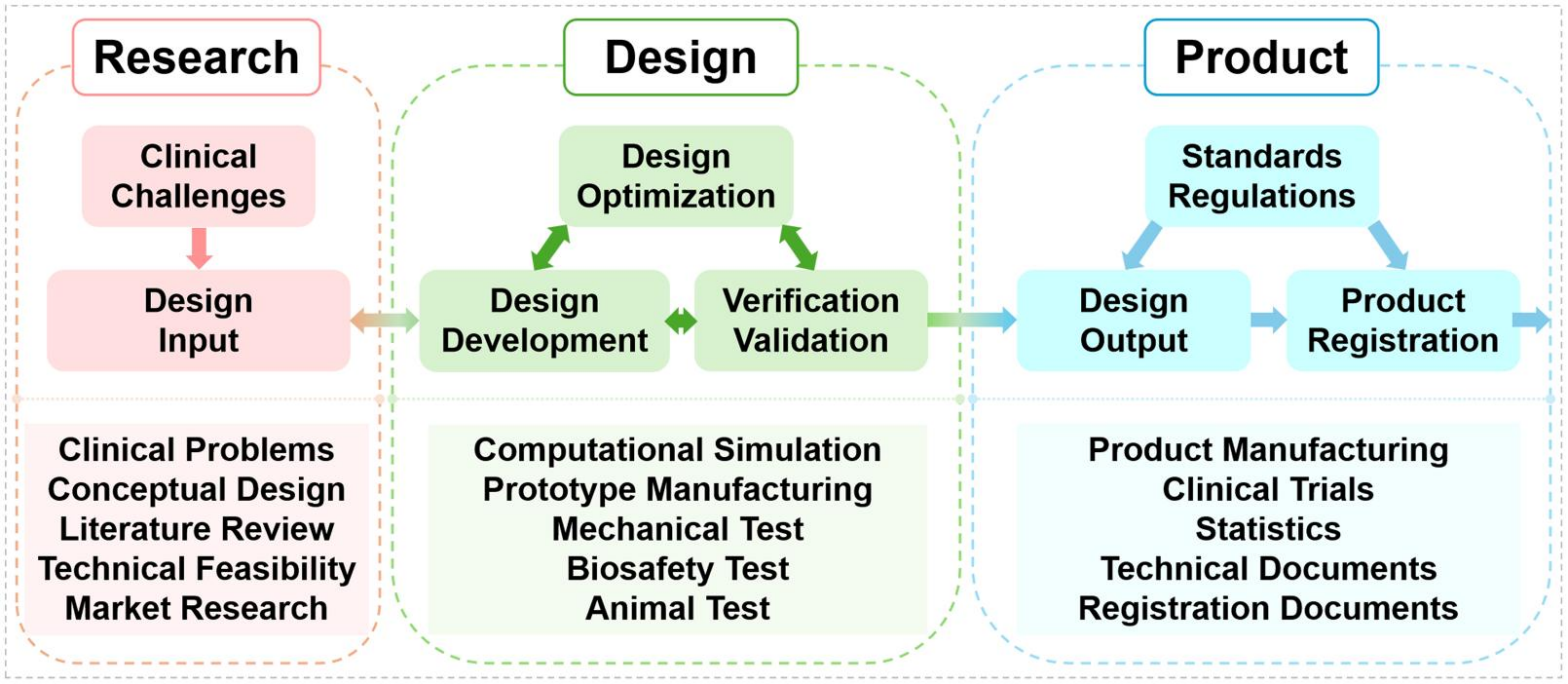
The development of the innovative Ti/Mg hybrid fixation system workflow.

The development of the innovative Ti/Mg hybrid fixation system follows a systematic workflow, as shown in Figure 7. It begins with the research phase to identify the clinical challenges and specific problems encountered in orthopedic applications. This phase includes a comprehensive literature review, in-depth scientific research and market research to guide the concept design and evaluate the technical feasibility of the proposed solution. The design phase focuses on the optimization and development of the hybrid fixation system. Design prototypes are developed through computational simulations to evaluate their biomechanical and biological performance under various physiological conditions. These designs were subsequently iteratively optimized based on the simulation results, resulting in the development of product prototypes for mechanical testing. Just as important is the patent application in this phase to protect intellectual property and for further technology transfer or clinical translation. Furthermore, the safety and efficacy of the innovative hybrid fixation system need to be evaluated through biosafety assessments and animal studies. The product phase includes the preparation for market launch. This includes conducting clinical trials to validate the overall performance of the innovative hybrid fixation system in human applications. Furthermore, it involves the preparation of comprehensive technical and regulatory documentation to ensure compliance with regulatory standards. Most importantly, the product must comply with relevant standards, regulations and laws throughout its lifecycle. In general, this structured method facilitates the effective translation of the Ti/Mg hybrid fixation system from concept to clinical application, supporting its clinical significance in enhancing fracture fixation and promoting bone healing as well as avoiding revision surgery.

Therefore, compared with conventional fracture fixation systems, the Ti/Mg hybrid fixation system offers a superior solution for fracture healing in osteoporotic patients. The Ti/Mg hybrid fixation system not only provides sufficient mechanical strength, but also contributes to fracture healing. Given these benefits, the application of this innovative hybrid technology may extend to a variety of fracture applications, where Mg degradation can contribute to bone healing and tissue regeneration while Ti or other alloys can provide sufficient mechanical strength to further enhance clinical outcomes [4].

## Conclusions

The application of biodegradable Mg in orthopedics is still in its early stage, while the boost increase of biodegradable Mg will be expected in the near future with intensified collaboration among material scientists, engineers and clinician scientists as well as regulatory bodies. This review systematically analyzes the orthopedic application prospects and the basic research of Mg, and then proposes the orthopedic application scheme of Mg in non-load skeletal bearing sites and load skeletal bearing sites based on the bedside needs. Finally, the translation pathway of Mg is outlined. For a comprehensive understanding of the orthopedic application prospects of Mg-based materials, the common biomaterials used in orthopedics, driving factors of orthopedic innovation, the physiology of Mg^2+^ are introduced. To achieve orthopedic application, modification of the performance of Mg as implantable metals and function of the degradation products of Mg *in vivo* are described. For the clinical needs of treating SAON in non-load skeletal bearing sites, Mg screws and Mg-based composite porous scaffolds (Mg/PLGA/TCP) have been developed. To utilize the beneficial biological effects of Mg degradation and overcome the weakness in mechanical stability for fracture fixation, the concept of developing Mg/Ti hybrid orthopedic implants is reported and updated. Finally, the translation process of Mg-based medical devices is stated to promote their clinical translation as innovative orthopedic implants. The gap between the materials applied in clinical orthopedics and materials in discovery and research is obvious due to the regulatory requirement for biosafety and treatment efficacy. This review provides a reference frame for the translation of novel materials and promotes the development of innovative orthopedic biomaterials for clinical applications. We hope the diversified, yet clinically relevant materials and medical devices will provide more choices for surgeons.
